# An integrated cognitive load–technology acceptance model for explaining behavioral intention to adopt Smart Physical Education Systems for extracurricular physical activity

**DOI:** 10.3389/fpsyg.2026.1811374

**Published:** 2026-05-11

**Authors:** Dan Dou, Bingyi Huang, Qiulu Chen, Xinjie Zhou, Cangen Wang

**Affiliations:** 1 School of Physical Education, Chongqing University of Posts and Telecommunications, Chongqing, China; 2 School of Foreign Languages, Southwest University of Political Science and Law, Chongqing, China

**Keywords:** behavioral intention, cognitive load theory, extracurricular physical activity, smart physical education systems, technology acceptance model

## Abstract

**Background:**

Smart Physical Education Systems are increasingly implemented in higher education institutions to promote undergraduates’ physical activity. However, sustained engagement with these systems remains limited. Although the Technology Acceptance Model explains technology acceptance evaluations and Cognitive Load Theory emphasizes constraints associated with cognitive processing, insufficient attention has been paid to how the allocation of cognitive resources influences undergraduates’ behavioral intention to adopt such systems. Integrating these perspectives may provide a more comprehensive understanding of the psychological mechanisms underlying technology adoption in digitally mediated exercise contexts.

**Methods:**

This study administered an electronic questionnaire survey to undergraduates from five higher education institutions that had implemented Smart Physical Education Systems, yielding 1,349 valid responses. Partial least squares structural equation modeling was employed to examine the structural relationships among the study variables. An integrated theoretical framework combining Cognitive Load Theory and the Technology Acceptance Model was adopted. The model comprised four technology acceptance constructs—perceived ease of use, perceived usefulness, attitude toward use, and behavioral intention—and three cognitive load dimensions—intrinsic, extraneous, and germane—to evaluate undergraduates’ behavioral intention to adopt Smart Physical Education Systems for extracurricular physical activity.

**Results:**

The study showed that hypothesized relationships were supported. Perceived ease of use, perceived usefulness, and attitude toward use predicted behavioral intention, with perceived ease of use demonstrating the strongest effect. Germane cognitive load exerted significant effects on perceived ease of use and perceived usefulness and indirectly enhanced behavioral intention. Conversely, intrinsic and extraneous cognitive load exerted significant effects on behavioral intention, underscoring the importance of cognitive load management in undergraduates’ adoption of Smart Physical Education Systems for extracurricular physical activity.

**Conclusion:**

Undergraduates’ behavioral intention to adopt Smart Physical Education Systems for extracurricular physical activity is shaped not only by perceived usefulness and perceived ease of use but also by the allocation of cognitive resources during system interaction. The integrated Cognitive Load–Technology Acceptance Model framework advances understanding of technology adoption in smart physical education contexts and provides both theoretical insights and practical guidance for the design of effective Smart Physical Education Systems.

## 1 Introduction

Smart Physical Education Systems (SPES) are digital platforms that integrate intelligent technologies into physical activity practice. Recent research has suggested that artificial intelligence can support innovation in physical education by linking instructional processes, feedback provision, and health-promotion functions (Cui et al., 2025). In university settings, SPES typically operate through campus-based platforms or mobile applications that support exercise-task delivery, activity check-in, performance recording, progress tracking, and feedback during undergraduates’ extracurricular physical activity. These systems may also connect with wearable devices to monitor indicators such as exercise duration, step counts, heart rate, or activity intensity, thereby helping undergraduates manage exercise beyond formal physical education classes (Johannes et al., 2024). Although SPES have become increasingly prominent tools for promoting physical activity in higher education settings (Huang, 2024; Oudah et al., 2024) their implementation does not necessarily lead to stable or sustained use.

Extracurricular physical activity (EPA), defined as self-initiated exercise undertaken outside formal classes, plays a critical role in fostering long-term exercise habits, psychological well-being, and overall health (Zhang, 2024). Compared with structured or compulsory activities, EPA offers greater flexibility and more closely aligns with undergraduates’ intrinsic motivations and behavioral preferences (Almeida et al., 2023). Because participation in EPA is shaped not only by behavioral opportunity but also by cognitive, motivational, and self-regulatory processes (Faílde-Garrido et al., 2022) subjective experiences and psychological perceptions during exercise are central to sustained participation (Ekkekakis, 2017). Although SPES offer a technological mechanism for supporting autonomous and sustained engagement in EPA, many undergraduates still engage with these systems only superficially and fail to integrate them into their regular exercise routines (Yao and Zhang, 2022). This phenomenon of gap between system availability and sustained use reflects a broader challenge in educational technology, where implementation does not necessarily translate into stable behavioral intention (Moghavvemi et al., 2013).

Technology Acceptance Model (TAM) provides a useful starting point for explaining why undergraduates may or may not adopt SPES for extracurricular physical activity. Originally proposed by Davis et al. (1989), TAM explains technology adoption as a psychological process in which users’ perceptions of a system shape their attitudes and subsequent behavioral intentions. TAM has become one of the most widely used frameworks for understanding technology acceptance and has demonstrated stable explanatory power across educational technology, digital health, human–computer interaction, and sport- and health-related contexts (Rosli et al., 2022; Lee et al., 2025). In the context of SPES, prior research has likewise shown that perceived ease of use (PEU) and perceived usefulness (PU) function as important antecedents of undergraduates’ continuance intention (Li et al., 2024). However, although TAM effectively explains whether users judge a system to be easy to use or useful, it says less about how these perceptions are formed during actual system interaction. This limitation is especially relevant in SPES contexts, where undergraduates must often engage with exercise tasks while simultaneously processing system information and feedback (Mayer, 2024). Thus, understanding SPES adoption may require moving beyond technology-related evaluations alone and considering the cognitive processes that underlie them (Ibili and Mark, 2019).

Cognitive Load Theory (CLT) offers a useful perspective for addressing this limitation. Originally proposed by Sweller (1988), CLT explains performance in terms of the limited capacity of working memory and distinguishes three types of cognitive load: intrinsic cognitive load (ICL), extraneous cognitive load (ECL), and germane cognitive load (GCL). ICL is associated with task complexity and element interactivity. ECL reflects unnecessary processing demands caused by suboptimal information presentation, whereas GCL captures cognitive resources deliberately allocated to schema construction and automation. These distinctions are highly relevant to SPES use because undergraduates often need to understand exercise tasks, interpret system feedback, and coordinate behavioral responses at the same time (Xu et al., 2024). In such situations, cognitive demands are not merely incidental; rather, they may become an important condition shaping. How the system is experienced and evaluated. CLT has been widely applied across educational psychology, human–computer interaction, digital learning environments, and research on the use of complex technological systems, and has become an important framework for explaining performance and behavioral responses in contexts characterized by high cognitive demands (Szulewski et al., 2021). Accordingly, CLT provides a theoretically relevant lens for understanding how different types of cognitive load may shape undergraduates’ perceptions of SPES and, ultimately, their behavioral intention to adopt the system for EPA.

Although the TAM and CLT have each been extensively applied to explain technology adoption and cognitive processing, relatively limited research has directly examined the interplay between technology-related evaluations and cognitive resource allocation during system use (Maričić et al., 2025). In cognitively demanding environments such as SPES, simultaneous task execution and system interaction may place additional demands on working memory (Hu et al., 2024). As a result, undergraduates’ behavioral intention may depend not only on whether the system is perceived as useful or easy to use, but also on how cognitive resources are consumed, disrupted, or directed during exercise-related interaction. A single-theory perspective is therefore unlikely to fully capture the psychological mechanisms underlying SPES adoption in EPA contexts (Benbasat and Barki, 2007; Zhong et al., 2025). To address this gap, the present study proposes and empirically tests an integrated Cognitive Load Theory–Technology Acceptance Model (CL–TAM). Rather than treating cognitive load as a peripheral background factor, this framework conceptualizes ICL, ECL, and GCL as distinct antecedents that shape PEU, PU, and ATU, which in turn influence BI. In this way, the study seeks to extend the explanatory scope of TAM by showing that technology acceptance is not determined solely by technology-related evaluations, but also by the cognitive processes through which those evaluations are formed. It thus provides a more specific theoretical account of SPES adoption in undergraduates’ EPA and offers a clearer basis for system design and implementation.

Building on the above rationale, this study develops and tests an integrated Cognitive Load Theory–Technology Acceptance Model (CL–TAM) to explain undergraduates’ behavioral intention to adopt SPES for extracurricular physical activity. Specifically, the model examines how ICL, ECL, and GCL shape PEU, PU, and ATU, and how these variables subsequently influence BI. By doing so, the study provides a more comprehensive account of SPES adoption in undergraduates’ EPA and offers implications for the design and implementation of smart physical education technologies.

## 2 Literature review and research hypotheses

### 2.1 Technology acceptance model

The Technology Acceptance Model (TAM), originally proposed by Davis et al. (1989), remains one of the most influential frameworks for explaining why individuals accept or reject technological systems. In its original formulation, perceived usefulness (PU) and perceived ease of use (PEU) were identified as two core beliefs shaping users’ attitudes toward a system and their subsequent behavioral intention to use it. Subsequent developments of the model further suggested that technology acceptance varies across contexts, including differences in voluntary versus mandatory use, user groups, and technology types (Montes, 2022). Meta-analytic evidence has likewise supported the general explanatory value of TAM across diverse usage settings, while also indicating that the magnitude of specific relationships may differ across contexts (King and He, 2006; Scherer and Teo, 2019). In educational settings, TAM has also been widely used to examine digital technology adoption and has demonstrated explanatory value in relation to acceptance-related beliefs and intentions (Xue et al., 2026).

The TAM is relevant to SPES use for EPA because SPES adoption involves undergraduates’ evaluations of whether the system is easy to use and useful for supporting exercise-related activities (Mackenbrock and Kleinert, 2026). Unlike technologies adopted in highly structured classroom environments, SPES for EPA are often embedded in flexible, self-directed, and often fragmented exercise situations. In such contexts, undergraduates may need to managing exercise plans, monitoring activity data, interpreting system feedback, and adjusting training behavior while interacting with the system (Zhong et al., 2025). Recent research on mobile learning outside the classroom suggests that perceived usefulness and perceived ease of use remain essential determinants of acceptance in voluntary and less structured technology-use environments (Liu et al., 2025). This point is important for the present study because EPA, like out-of-class mobile learning, is characterized by greater autonomy and weaker external compulsion (Nurmi et al., 2016).

Therefore, technology acceptance in this context may depend not only on perceptions of system functionality, but also on whether the system can be integrated into ongoing self-directed exercise practice (Yousaf et al., 2021; Ebadi and Raygan, 2023).

A substantial body of research has shown that PEU positively predicts PU, and this relationship has remained one of the most stable pathways in TAM research since the original model. When a system can be learned and operated with relatively little effort, users are more likely to recognize its instrumental value and practical benefits. This pattern has been supported not only in general technology acceptance research but also in educational technology contexts, where ease of use contributes to more favorable evaluations of usefulness (Mastour et al., 2025). Related evidence from health and fitness app research likewise suggests that perceived ease of use enhances perceived usefulness and, through it, strengthens continued engagement with the technology (Cho et al., 2020; Samadbeik et al., 2023). In the context of SPES for EPA, this suggests that undergraduates who perceive the system as easy to operate may be more likely to regard it as useful for supporting their exercise practice. Accordingly, the following hypothesis is proposed:

*H1*: Undergraduates’ PEU of SPES for EPA positively affects PU.

The PEU is also expected to positively influence attitude toward use (ATU). Prior TAM research has consistently suggested that systems requiring less effort tend to generate smoother and more favorable usage experiences, thereby facilitating more positive user attitudes. Although the strength of the PEU–ATU relationship may vary across technologies and user groups, educational research has repeatedly shown that ease-related beliefs remain important in shaping users’ evaluative orientation toward technology (Çetin, 2020; Liesa-Orús et al., 2023). In physical education settings, recent research likewise indicates that ease-related perceptions and lower complexity barriers are conducive to more favorable acceptance of digital media (Wang et al., 2022; Mackenbrock and Kleinert, 2026). In the context of SPES for EPA, a system that is cumbersome or difficult to navigate may interfere with ongoing exercise practice and reduce positive evaluations, whereas a system that feels accessible and manageable is more likely to foster favorable attitudes toward use (Drehlich et al., 2020). Therefore, the following hypothesis is proposed:

H2: Undergraduates’ PEU of SPES for EPA positively affects ATU.

In addition, PEU may directly influence behavioral intention (BI), especially in contexts where use is largely discretionary (Zhang and Yu, 2022). In self-directed contexts, convenience and low operational burden may affect users’ willingness to adopt a system more directly, particularly because users can more easily disengage from technologies when usage becomes effortful, confusing, or disruptive (Pan, 2020; Angosto et al., 2023; Harris and Rogers, 2025). Recent work on mobile learning outside the classroom similarly shows that PEU remains an important determinant of acceptance in voluntary, out-of-class usage contexts (Berkowsky et al., 2017; Bergman et al., 2020). Moreover, studies on health and fitness apps suggest that PEU not only improves cognitive appraisals of usefulness but also supports continued intention to use such technologies (Yan et al., 2021). Given that SPES for EPA are often used during flexible and non-compulsory exercise participation, undergraduates’ intention to adopt the system may depend (Lai et al., 2022), at least in part, on whether it can be used conveniently and effortlessly. Hence, the following hypothesis is proposed:

*H3*: Undergraduates’ PEU of SPES for EPA positively affects BI.

The PU is another core determinant in TAM because users are more likely to accept a technology when they believe it can improve task performance, efficiency, or outcomes. Since the original TAM, perceived usefulness has often been identified as an important predictor of both favorable evaluations and intention to use a system. This pattern has been supported not only in general technology acceptance research but also in educational contexts, where PU has been shown to play an important role in shaping acceptance-related outcomes (Hayadi and Hariguna, 2025). Similar evidence has also been reported in smart wearable and sport-related technology research. For example, research on smart wearable devices has shown that usefulness is one of the key factors shaping users’ intention to continue using such technologies (Park, 2020). In the context of SPES, usefulness should be understood not as an abstract technical attribute, but as undergraduates’ judgment that the system can meaningfully support exercise planning, activity monitoring, performance feedback, and goal attainment (Zulkifli and Danis, 2022). When undergraduates perceive such value in the system, they may be more likely to develop favorable attitudes toward it and stronger intention to adopt it for extracurricular physical activity. On this basis, the following hypotheses are proposed:

*H4*: Undergraduates’ PU of SPES for EPA positively affects ATU.

*H5*: Undergraduates’ PU of SPES for EPA positively affects BI.

Finally, attitude toward use (ATU) is commonly treated as a proximal antecedent of behavioral intention in TAM-based research. Although some extended acceptance models have reduced the prominence of attitude, prior research has argued that ATU still deserves explicit attention because it captures users’ overall evaluative orientation toward adopting a technology (Kim et al., 2009). Empirical evidence in higher education has likewise shown that students’ attitudes are significantly associated with their intention to use digital learning systems (Hussein, 2017). Similar findings have also been reported in health- and sport-related technology contexts, where users’ attitudes have been found to significantly predict intention to adopt wearable fitness technologies (Lee and Lee, 2018). In the context of SPES for EPA, extracurricular physical activity is characterized by a relatively high degree of autonomy and self-regulation (Barkoukis et al., 2021). Under such conditions, undergraduates may be more likely to incorporate SPES into their ongoing exercise practice when they develop a generally favorable attitude toward using the system (Song et al., 2018). Therefore, the following hypothesis is proposed:

*H6*: Undergraduates’ ATU toward SPES for EPA positively affects BI.

### 2.2 Cognitive load theory

While TAM explains how evaluative beliefs shape attitude and behavioral intention, it does not fully specify the cognitive conditions under which such evaluations emerge. In the context of SPES for extracurricular physical activity, undergraduates may need to not only interact with the system but also process exercise tasks, interpret feedback, and regulate training behavior. Accordingly, Cognitive Load Theory provides an important complementary perspective by clarifying how different forms of cognitive load may facilitate or constrain users’ perceptions of ease of use, usefulness, and overall attitudes toward the system. Originally proposed by Sweller (1988), CLT explains learning and performance in terms of the limited capacity of working memory and the allocation of cognitive resources. CLT classifies cognitive load into three types: ICL, ECL, and GCL (Paas et al., 2003). ICL primarily stems from the inherent complexity of the task itself and the degree of interaction among multiple elements that must be processed simultaneously (Chen et al., 2023). Prior research generally suggests that when intrinsic load is high, a larger share of limited cognitive resources must be devoted to task execution itself, leaving fewer resources available for evaluative and strategic processing (Van Merriënboer and Sweller, 2010). In technology-supported task environments, this pattern may reduce users’ ability to form favorable judgments about the usefulness and value of the supporting system.

In this study, ICL specifically refers to the complexity associated with using SPES to conduct EPA and the demands of integrating multiple task components. Undergraduates must concurrently comprehend exercise plans, master movement techniques, regulate training intensity, and apply self-monitoring strategies while making ongoing judgments and adjustments during actual practice (Carlier and Delevoye-Turrell, 2022). This imbalance in resource allocation may undermine users’ perceptions of system effectiveness and usefulness, thereby weakening their overall evaluation of the system (Fink et al., 2023). In the context of SPES for EPA, this mechanism suggests that higher intrinsic cognitive load may undermine undergraduates’ perceptions of usefulness and their attitudes toward the system. Accordingly, the following hypotheses are proposed:

*H7*: undergraduates’ ICL of SPES for EPA negatively affects ATU.

*H8*: undergraduates’ ICL of SPES for EPA negatively affects PU.

The ECL arises from unnecessary processing demands imposed by inappropriate information presentation, complex interface design, or redundant interaction procedures (Liang et al., 2023). In the context of SPES, such load may be generated by disorganized layouts, cumbersome operational pathways, excessive feedback information, or unclear instructions. These design deficiencies divert cognitive resources toward system-related operations rather than exercise-relevant activities, thereby limiting users’ ability to focus on physical activity itself. Prior research has shown that reducing extraneous load through clearer information presentation and more streamlined interaction procedures can improve users’ perceptions of ease of use and overall system evaluation (Ibili and Mark, 2019; Hussein et al., 2022). By contrast, elevated ECL may make system operation feel unnecessarily effortful, thereby reducing PEU, while also contributing to frustration and negative usage experiences that weaken attitudes toward the system (Turetken et al., 2019). Accordingly, the following hypotheses are proposed:

*H9*: undergraduates’ ECL of SPES for EPA negatively affects PEU.

*H10*: undergraduates’ ECL of SPES for EPA negatively affects ATU.

The GCL emphasizes the cognitive resources that learners deliberately invest to facilitate schema construction and the automation of knowledge structures (Sweller et al., 1998; Debue and Van De Leemput, 2014). Importantly, cognitive load should not be viewed as uniformly detrimental in technology adoption contexts. Unlike intrinsic and extraneous load, germane cognitive load reflects productive cognitive investment in understanding, schema construction, and task-relevant processing (Klepsch and Seufert, 2020).

In this study, GCL specifically refers to undergraduates’ purposeful cognitive engagement in understanding SPES feedback, interpreting exercise strategies, and developing stable operational routines and training rules. As effective processing increases, undergraduates may be more likely to form clear mental representations of the system functional logic and interaction pathways, thereby enhancing their subjective perceptions of operational fluency and controllability, which may be reflected in higher perceived ease of use (Ibili and Mark, 2019). At the same time, GCL may strengthen users’ sense of understanding and control over the training process, thereby fostering more positive evaluations of the system (El-Gayar and Elnoshokaty, 2023). Moreover, when undergraduates invest greater cognitive effort in interpreting exercise data, optimizing training plans, and internalizing motor skills, they may be more likely to recognize the instrumental value of SPES in improving exercise efficiency, enhancing performance, and supporting goal attainment, thereby increasing perceived usefulness (Alshawmar et al., 2025).

Thus, GCL is treated here not as a burden, but as a facilitating cognitive condition that may strengthen technology-related appraisals. Based on the above theoretical reasoning and supporting empirical evidence, the following hypotheses are proposed:

*H11*: Undergraduates’ GCL of SPES for EPA positively affects PEU.

*H12*: Undergraduates’ GCL of SPES for EPA positively affects ATU.

*H13*: Undergraduates’ GCL of SPES for EPA positively affects PU.

**Figure 1:**
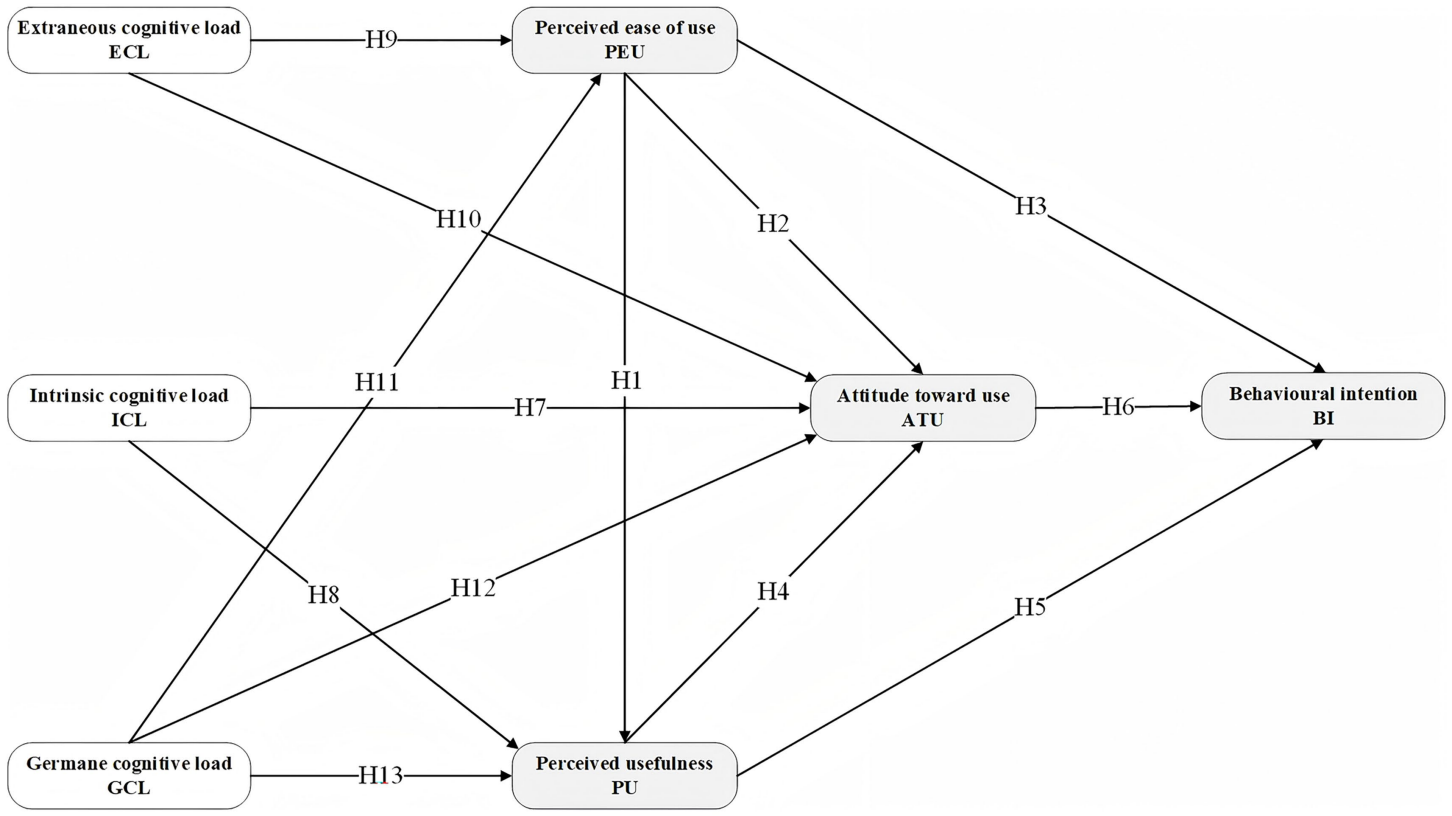
Hypothetical model.

Based on hypotheses H1 to H13, this study proposes an integrated CL–TAM conceptual model that integrates CLT and the TAM. The structure of the proposed research model is illustrated in Figure 1.

## 3 Methods

### 3.1 Participants and data collection

The study recruited undergraduate students from five universities in China that had implemented SPES, namely Chongqing University of Posts and Telecommunications, Chongqing Technology and Business University, Sichuan International Studies University, Chongqing Second Normal University, and Yangtze Normal University. Although the specific platform names varied across the five universities, their core functions were comparable. In this study, participants evaluated SPES based on the systems already deployed in their universities for extracurricular physical activity management and support. These systems generally included exercise-task assignment, activity check-in, exercise-data recording, progress tracking, feedback provision, and, in some cases, connectivity with wearable devices. All participants had prior exposure to the systems used in their universities, and their responses were based on actual use experience in these institutional contexts. Participants’ responses were therefore anchored in their actual use experience with these systems rather than in hypothetical judgments about smart sports technologies in general.

The questionnaires were organized and distributed by the research team through both online and offline formats to enhance sample coverage and reduce the limitations associated with a single survey mode. The online survey facilitated broader and more efficient access to potential respondents, whereas the paper-based survey was administered in person to improve participation and reduce potential selection bias. Before completing the questionnaire, all participants were informed of the purpose of the study and voluntarily provided informed consent. Ethical approval was obtained from the Research Ethics Committee of Chongqing University of Posts and Telecommunications prior to data collection (Approval No. 202512150101389).

Participants were eligible for inclusion if they were full-time undergraduates enrolled in one of the five participating universities and had prior experience using SPES in the context of extracurricular physical activity. During data screening, questionnaires were excluded if they were incomplete, failed attention-check items, or showed inconsistent responses on reverse-coded items or other logically contradictory response patterns. Following the survey quality assessment framework proposed by Borzemski et al. (2019) the dataset was further screened to ensure response validity and internal consistency. A total of 1,506 questionnaires were collected, and 157 invalid responses were removed during data cleaning. The final sample therefore consisted of 1,349 valid responses for subsequent statistical analyses and structural model testing. Table 1 presents the demographic characteristics of the participants.

**Table 1:** Demographic characteristics of participants.

Variable	Category	*n*	%
Gender	Male	729	54.0
	Female	620	46.0
University	Chongqing University of Posts and Telecommunications	798	59.2
	Chongqing Technology and Business University	167	12.4
	Sichuan International Studies University	126	9.3
	Chongqing Second Normal University	135	10.0
	Yangtze Normal University	123	9.1
Grade	First-year	455	33.7
	Second year	412	30.5
	Third-year	327	24.2
	Fourth-year	155	11.6
Discipline	Engineering	696	51.6
	Literature	136	10.1
	Science	76	5.6
	Law	25	1.9
	Management	135	10.0
	Economics	138	10.2
	Arts	86	6.4
	Education	57	4.2

### 3.2 Measures

A structured questionnaire was used as the primary measurement instrument in this study. The items were adapted and refined from previously validated scales to ensure contextual relevance and measurement rigor (Hoque and Sorwar, 2017), enabling a systematic assessment of undergraduates’ perceptions and behavioral responses during SPES use for extracurricular physical activity. The measured constructs included the core TAM variables—perceived usefulness (PU), perceived ease of use (PEU), attitude toward use (ATU), and behavioral intention (BI)—and the three dimensions of cognitive load theory—intrinsic cognitive load (ICL), extraneous cognitive load (ECL), and germane cognitive load (GCL). The sources and operationalization of all measurement items are reported in Table 2.

**Table 2:** Constructs and their sources.

Constructs	Sources
PU	Davis et al. (1989), Teo et al. (2008), Teo and Noyes (2011), Teo (2009), Lavidas et al. (2020), Al-Emran et al. (2021), Lin and Yu (2023), Tan et al. (2024), Albayati (2024)
PEU	Davis et al. (1989), Teo et al. (2008), Teo and Noyes (2011), Lavidas et al. (2020), Al-Emran et al. (2021), Lin and Yu (2023), Tan et al. (2024)
ATU	Teo (2009), Teo and Noyes (2011), Tan et al. (2024)
BI	Venkatesh et al. (2003), Teo (2009), Teo and Noyes (2011), Duong et al. (2023), Wang et al. (2025)
ICL	Bratfisch et al. (1972), Sweller et al. (1998), Krieglstein et al. (2023), Huang et al. (2025)
ECL	Bratfisch et al. (1972), Sweller et al. (1998), Krieglstein et al. (2023), Huang et al. (2025)
GCL	Bratfisch et al. (1972), Sweller et al. (1998), Krieglstein et al. (2023), Huang et al. (2025)

During item adaptation, the original wording of the scales was revised to fit the context of undergraduates’ use of SPES for extracurricular physical activity. For example, the item “Behavioral recognition functions in the SPES, such as facial recognition and location-based check-in, increase my burden when using the system” was adapted from Huang et al. (2025) and further revised for the present study. This wording adjustment was intended to capture the additional burden imposed by SPES-related operational requirements, while remaining conceptually consistent with the broader understanding of ECL in earlier research (Bratfisch et al., 1972; Sweller et al., 1998).

All items were rated on an 11-point Likert scale. This format was selected to capture subtle differences in undergraduates’ perceptions of SPES use for extracurricular physical activity and to provide greater response differentiation across the multiple latent constructs examined in this study. Given the large sample size and the use of PLS-SEM, the 11-point scale was considered appropriate for generating more fine-grained measurement information. This choice is also supported by previous methodological research suggesting that 11-point Likert scales may offer greater sensitivity and distributional properties closer to interval-level scaling and normality than shorter formats (Leung, 2011).

### 3.3 Data analysis

SmartPLS 4.0 was employed to estimate the proposed CL–TAM model. PLS-SEM was selected because the present study did not focus on a single bivariate relationship; instead, it examined a theoretically integrated mechanism in which three dimensions of cognitive load (ICL, ECL, and GCL) were linked to behavioral intention both directly and indirectly through perceived ease of use, perceived usefulness, and attitude toward use. Accordingly, the analytical task required a method capable of modeling multiple latent constructs and testing a network of interdependent structural paths within one unified framework. In addition, because the study aimed to explain and predict undergraduates’ intention to adopt Smart Physical Education Systems for extracurricular physical activity, PLS-SEM provided an appropriate analytic approach for simultaneously evaluating the measurement properties of the constructs and the predictive relationships specified in the structural model.

The data analysis was structured around the logic of the integrated CL–TAM model. First, the measurement model was assessed by examining the reliability and validity of the seven latent constructs. Second, the hypothesized structural relationships were tested by estimating the path coefficients and their statistical significance through bootstrapping. Specifically, this step examined whether ICL, ECL, and GCL were differentially associated with PEU, PU, and ATU, and whether these variables were also associated with BI. Third, the explanatory and predictive performance of the integrated model was examined using the coefficient of determination (*R*^2^), effect size (*f*^2^), and predictive relevance (*Q*^2^) for the endogenous constructs, particularly PEU, PU, ATU, and BI.

## 4 Results

### 4.1 Measurement model

#### 4.1.1 Reliability

Reliability was assessed at both the indicator and construct levels. Indicator reliability was first examined using the outer loadings of the observed indicators. As shown in Table 3, all outer loadings ranged from 0.708 to 0.919, exceeding the recommended threshold of 0.70 (Preacher and MacCallum, 2003), thereby indicating satisfactory indicator reliability.

**Table 3:** Construct reliability and validity.

Latent variable	Item	Outer loadings	Cronbach’s alpha	Composite reliability (rho_a)	Composite reliability (rho_c)	Average variance extracted (AVE)
ATU	ATU1	0.736	0.846	0.863	0.885	0.562
	ATU2	0.788				
	ATU3	0.824				
	ATU4	0.712				
	ATU5	0.708				
	ATU6	0.724				
BI	BI1	0.902	0.947	0.947	0.959	0.825
	BI2	0.919				
	BI3	0.919				
	BI4	0.911				
	BI5	0.892				
ECL	ECL1	0.774	0.929	0.947	0.941	0.666
	ECL2	0.776				
	ECL3	0.866				
	ECL4	0.843				
	ECL5	0.829				
	ECL6	0.823				
	ECL7	0.809				
	ECL8	0.807				
GCL	GCL1	0.795	0.885	0.886	0.916	0.686
	GCL2	0.789				
	GCL3	0.860				
	GCL4	0.858				
	GCL5	0.835				
ICL	ICL1	0.859	0.892	0.896	0.918	0.652
	ICL2	0.852				
	ICL3	0.844				
	ICL4	0.804				
	ICL5	0.758				
	ICL6	0.717				
PEU	PEU1	0.741	0.736	0.736	0.835	0.558
	PEU2	0.745				
	PEU3	0.768				
	PEU4	0.733				
PU	PU1	0.867	0.888	0.890	0.923	0.749
	PU2	0.853				
	PU3	0.890				
	PU4	0.851				

Construct reliability was subsequently evaluated using Cronbach’s alpha and composite reliability indices (rho_a and rho_c). Values above 0.70 are generally considered acceptable (Kilic, 2016). Cronbach’s alpha values ranged from 0.736 to 0.947, rho_a values from 0.736 to 0.947, and rho_c values from 0.835 to 0.959. All values exceeded the recommended threshold, indicating satisfactory internal consistency across all constructs.

Overall, the measurement model demonstrated adequate reliability and provided a sound basis for subsequent assessments of convergent validity, discriminant validity, and the structural model.

#### 4.1.2 Validity

Convergent validity was assessed using the average variance extracted (AVE). Values of 0.50 or higher indicate that a construct explains more than half of the variance of its indicators (Qin et al., 2024). As shown in Table 3, all AVE values ranged from 0.558 to 0.825, exceeding the recommended threshold and thereby indicating satisfactory convergent validity. Discriminant validity was subsequently assessed using the Fornell–Larcker criterion, which compares the square root of the AVE of each construct with the inter-construct correlations. According to Fornell and Larcker (1981) discriminant validity is established when the square root of the AVE for a construct exceeds its correlations with all other constructs. As shown in Table 4, all diagonal values were greater than the corresponding inter-construct correlations, thereby satisfying the Fornell–Larcker criterion and indicating satisfactory discriminant validity.

**Table 4:** Fornell–Larcker.

Construct	ATU	BI	ECL	GCL	ICL	PEU	PU
ATU	0.75						
BI	0.756	0.908					
ECL	−0.597	−0.424	0.816				
GCL	0.633	0.697	−0.386	0.828			
ICL	−0.654	−0.453	0.719	−0.385	0.807		
PEU	0.596	0.718	−0.413	0.624	−0.374	0.747	
PU	0.717	0.697	−0.381	0.561	−0.45	0.543	0.866

The heterotrait–monotrait ratio (HTMT) is a more recently proposed method for assessing discriminant validity. Its underlying principle involves comparing the correlations between indicators of different constructs with the correlations among indicators within the same construct to determine whether latent variables can be empirically distinguished from one another. In general, HTMT values below 0.85 are considered indicative of strong discriminant validity, although some studies suggest that values below 0.90 may also be acceptable (Ab Hamid et al., 2017). As shown in Table 5, all HTMT values among the latent constructs in this study were below 0.90, demonstrating satisfactory discriminant validity. Notably, these values consistently fell within the recommended thresholds, further confirming the robustness of the model’s discriminant validity.

**Table 5:** Discriminant validity-HTMT.

Construct	ATU	BI	ECL	GCL	ICL	PEU	PU
ATU							
BI	0.807						
ECL	0.656	0.433					
GCL	0.711	0.761	0.410				
ICL	0.771	0.488	0.779	0.430			
PEU	0.725	0.860	0.485	0.773	0.458		
PU	0.789	0.757	0.395	0.631	0.493	0.670	

#### 4.1.3 Collinearity assessment

To enhance the robustness of the measurement model and reduce the potential risk of multicollinearity, variance inflation factors (VIF) were examined for all constructs. As shown in Table 6, the VIF values for the latent variables ranged from 1.175 ECL to 2.350 ATU, all of which were below the widely accepted threshold of 3.0. Results indicate that multicollinearity was not a concern and that the constructs were sufficiently distinct from one another, thereby providing additional support for the reliability and validity of the measurement model.

**Table 6:** Collinearity statistics (VIF).

Construct	ATU	BI	ECL	GCL	ICL	PEU	PU
ATU		2.35					
BI							
ECL	2.186					1.175	
GCL	1.881					1.175	1.713
ICL	2.242						1.216
PEU	1.864	1.619					1.696
PU	1.727	2.150					

### 4.2 Structural model

The coefficient of determination *R*^2^ value is a key statistical indicator in structural model evaluation, reflecting the proportion of variance in endogenous variables that can be explained by the model’s predictors (Nakagawa and Schielzeth, 2013). As shown in Table 7, the *R*^2^ values were 0.717 for ATU, 0.707 for BI, 0.424 for PEU, and 0.420 for PU. These results indicate that the proposed model demonstrates satisfactory explanatory power for all endogenous constructs. Moreover, the adjusted *R*^2^ values for ATU, BI, PEU, and PU were highly consistent with their corresponding *R*^2^, suggesting that the model’s explanatory power was not artificially inflated by the inclusion of additional predictors. This consistency further supports the robustness of the model specification and the appropriateness of the selected exogenous variables. Overall, the results indicate that the complexity of the model was properly controlled, thereby avoiding both model under-specification and the risk of overfitting due to excessive structural complexity.

**Table 7:** Results of the *R*^2^.

Constructs	*R*-square	*R*-square adjusted
ATU	0.717	0.716
BI	0.707	0.706
PEU	0.424	0.423
PU	0.420	0.419

The *Q*^2^ value was used to assess the predictive relevance of the structural model for the endogenous constructs (Consonni et al., 2010). In this study, the endogenous variables included ATU (*Q*^2^ = 0.395), BI (*Q*^2^ = 0.579), PEU (*Q*^2^ = 0.234), and PU (*Q*^2^ = 0.311). As shown in Table 8, all *Q*^2^ values were greater than zero, indicating that the model demonstrates adequate predictive relevance and satisfactory predictive capability for the key endogenous constructs.

**Table 8:** Results of the *Q*^2^.

Constructs	SSO	SSE	*Q* ^2^
ATU	8,094	4893.04	0.395
BI	6745.000	2837.674	0.579
PEU	5396.000	4132.971	0.234
PU	5396.000	3717.295	0.311

The *F*^2^ value assesses effect size by indicating each exogenous variable’s incremental contribution to the variance explained in endogenous constructs. Comparing *F*^2^ values across paths helps identify the most influential predictors. Values of 0.02, 0.15, and 0.35 indicate small, medium, and large effects. As shown in Table 9, ATU (*F*^2^ = 0.197) and PEU (*F*^2^ = 0.296) exhibited medium effects on BI, indicating that they are the primary determinants of undergraduates’ BI. In contrast, PU showed a relatively small effect on BI (*F*^2^ = 0.083). Meanwhile, PU demonstrated a medium effect on ATU (*F*^2^ = 0.281), suggesting that perceived usefulness plays an important role in attitude formation. GCL exerted the largest effect on PEU (*F*^2^ = 0.441), reaching a large effect size and highlighting its critical role in enhancing perceived ease of use. ICL showed small-to-medium effects on ATU (*F*^2^ = 0.115) and PU (*F*^2^ = 0.077), whereas ECL had generally weak effects on related constructs (*F*^2^ ranging from 0.031 to 0.060). Overall, ATU, PEU, and GCL contributed most substantially to the model’s explanatory power. Although the remaining variables showed smaller effects, they remain integral to understanding undergraduates’ intention to use SPES for EPA.

**Table 9:** Results of the *F*^2^ of the model.

Constructs	ATU	BI	ECL	GCL	ICL	PEU	PU
ATU		0.197					
BI							
ECL	0.031					0.060	
GCL	0.073					0.441	0.094
ICL	0.115						0.077
PEU	0.024	0.296					0.071
PU	0.281	0.083					

### 4.3 Structural model assessment

#### 4.3.1 Path coefficients

Path coefficients were examined to assess the direction and strength of the hypothesized relationships among the latent variables. As shown in Table 10, all structural paths were statistically significant (*p* < 0.00 1), thereby supporting all 13 proposed hypotheses. Within the TAM framework, PEU positively predicted PU (*β* = 0.265, *p* < 0.001), ATU (*β* = 0.113, *p* < 0.001), and BI (*β* = 0.375, *p* < 0.001). PU also showed significant positive effects on ATU (*β* = 0.371, *p* < 0.001) and BI (*β* = 0.229, *p* < 0.001), while ATU positively predicted BI (*β* = 0.368, *p* < 0.001). Within the cognitive load framework, ICL negatively affected ATU (*β* = −0.270, *p* < 0.001) and PU (*β* = −0.233, *p* < 0.001), ECL negatively affected PEU (*β* = −0.202, *p* < 0.001) and ATU (*β* = −0.139, *p* < 0.001), whereas GCL positively influenced PEU (*β* = 0.546, *p* < 0.001), PU (*β* = 0.306, *p* < 0.001), and ATU (*β* = 0.197, *p* < 0.001).

**Table 10:** Path coefficients.

Hypotheses	Path	Original sample	Sample mean	Standard deviation	*T* statistics	*p*-values	Results
H1	PEU → PU	0.265	0.265	0.032	8.323	*p* < 0.001	Supported
H2	PEU → ATU	0.113	0.113	0.023	4.906	*p* < 0.001	Supported
H3	PEU → BI	0.375	0.374	0.026	14.637	*p* < 0.001	Supported
H4	PU → ATU	0.371	0.371	0.023	16.348	*p* < 0.001	Supported
H5	PU → BI	0.229	0.23	0.027	8.613	*p* < 0.001	Supported
H6	ATU → BI	0.368	0.368	0.025	14.593	*p* < 0.001	Supported
H7	ICL → ATU	−0.27	−0.27	0.026	10.563	*p* < 0.001	Supported
H8	ICL → PU	−0.233	−0.233	0.023	10.292	*p* < 0.001	Supported
H9	ECL → ATU	−0.139	−0.139	0.023	6.038	*p* < 0.001	Supported
H10	ECL → PEU	−0.202	−0.202	0.025	8.19	*p* < 0.001	Supported
H11	GCL → ATU	0.197	0.197	0.022	9.064	*p* < 0.001	Supported
H12	GCL → PEU	0.546	0.546	0.024	23.158	*p* < 0.001	Supported
H13	GCL → PU	0.306	0.306	0.033	9.415	*p* < 0.001	Supported

Several findings deserve particular attention. PEU exerted a stronger direct effect on BI than PU and was also slightly stronger than ATU, suggesting that ease of use was the most influential proximal determinant of undergraduates’ intention to adopt SPES. This pattern departs from much of the traditional TAM literature, in which PU is often regarded as the dominant predictor of behavioral intention. In the context of extracurricular physical activity, however, undergraduates may place greater emphasis on whether SPES can be used conveniently and effortlessly in flexible, self-directed exercise settings.

The path from GCL to PEU (*β* = 0.546) was the strongest direct effect in the model. This result suggests that PEU may arise not only from interface simplicity or system functionality, but also from whether the system facilitates meaningful cognitive engagement and schema construction. In this sense, PEU in SPES adoption appears to be shaped partly by cognitive processing conditions rather than by technological characteristics alone, which further supports the integration of CLT and TAM.

A further notable finding is that the negative effect of ICL on ATU was substantially stronger than that of ECL. This result indicates that the inherent complexity of extracurricular exercise tasks may undermine undergraduates’ attitudes toward SPES more strongly than interface burden or redundant information. Thus, reducing interface-related burden alone may not be sufficient if the exercise tasks themselves remain cognitively demanding.

#### 4.3.2 Indirect effects

Indirect effects were examined to determine whether the influences of cognitive load on outcome variables operated through the mediating mechanisms specified in the integrated CL–TAM model. As shown in Table 11, all 11 indirect effects were statistically significant (*p* < 0.001), indicating that cognitive load influenced BI primarily through PEU, PU, and ATU rather than through isolated direct pathways.

**Table 11:** Indirect effects.

Path	Original sample	Sample mean	Standard deviation	*T* statistics	*P*-values
PU → BI	0.136	0.136	0.012	11.772	*p* < 0.001
PEU → BI	0.138	0.139	0.015	9.09	*p* < 0.001
PEU → ATU	0.098	0.098	0.013	7.301	*p* < 0.001
ICL → BI	−0.184	−0.184	0.015	12.582	*p* < 0.001
ICL → ATU	−0.086	−0.086	0.010	8.679	*p* < 0.001
GCL → PU	0.145	0.145	0.019	7.736	*p* < 0.001
GCL → BI	0.465	0.465	0.018	25.196	*p* < 0.001
GCL → ATU	0.229	0.229	0.018	13.015	*p* < 0.001
ECL → PU	−0.054	−0.054	0.009	5.865	*p* < 0.001
ECL → BI	−0.155	−0.155	0.015	10.209	*p* < 0.001
ECL → ATU	−0.043	−0.043	0.007	5.92	*p* < 0.001

Among the TAM variables, PEU exerted significant positive indirect effects on ATU (*β* = 0.098, *p* < 0.001) and BI (*β* = 0.138, *p* < 0.001), while PU showed a significant positive indirect effect on BI (*β* = 0.136, *p* < 0.001). These results indicate that the influence of ease of use extends beyond its direct contribution to intention and is further transmitted through more favorable usefulness perceptions and attitudes.

More importantly, GCL displayed the strongest indirect influence pattern in the model. It exerted significant positive indirect effects on PU (*β* = 0.145, *p* < 0.001), ATU (*β* = 0.229, *p* < 0.001), and especially BI (*β* = 0.465, *p* < 0.001). The magnitude of this indirect effect on BI exceeded that of all other variables, suggesting that germane cognitive load functions as a central mechanism linking cognitive processing to technology adoption. Rather than serving as a peripheral antecedent, GCL appears to enhance behavioral intention by reinforcing the core TAM sequence of PEU, PU, and ATU.

By contrast, both ICL and ECL showed significant negative indirect effects. ICL negatively affected ATU (*β* = −0.086, *p* < 0.001) and BI (*β* = −0.184, *p* < 0.001), whereas ECL negatively affected PU (*β* = −0.054, *p* < 0.001), ATU (*β* = −0.043, *p* < 0.001), and BI (*β* = −0.155, *p* < 0.001). Notably, the negative indirect effect of ICL on BI was stronger than that of ECL, further indicating that task-related cognitive demands may be more detrimental to sustained adoption than interface-related burden.

#### 4.3.3 Total effects

Total effects were examined to identify the most influential predictors of undergraduates’ behavioral intention to adopt SPES. As shown in Table 12, all total effects were statistically significant (*p* < 0.001). Within the TAM framework, PEU exhibited the largest total effect on BI (*β* = 0.513, *p* < 0.001), exceeding the effects of ATU (*β* = 0.368, *p* < 0.001) and PU (*β* = 0.366, *p* < 0.001). This result further confirms that ease of use is the most important determinant of undergraduates’ continued intention to adopt SPES. Moreover, the large total effect of PEU indicates that its influence extends beyond a direct pathway to BI and is further reinforced through its positive effects on PU and ATU. Within the CLT framework, GCL also demonstrated a substantial positive total effect on BI (*β* = 0.465, *p* < 0.001), whereas ICL (*β* = −0.184, *p* < 0.001) and ECL (*β* = −0.155, *p* < 0.001) both reduced BI. More importantly, the magnitude of the total effect of GCL was considerably larger than the negative total effects of ICL and ECL. This finding suggests that promoting meaningful cognitive engagement may be more effective than merely reducing cognitive burden.

**Table 12:** Total effects.

Path	Original sample	Sample mean	Standard deviation	*T* statistics	*P*-values
PEU → BI	0.513	0.513	0.025	20.124	*p* < 0.001
PU → BI	0.366	0.366	0.026	14.238	*p* < 0.001
ATU → BI	0.368	0.368	0.025	14.593	*p* < 0.001
ECL → BI	−0.155	−0.155	0.015	10.209	*p* < 0.001
ICL → BI	−0.184	−0.184	0.015	12.582	*p* < 0.001
GCL → BI	0.465	0.465	0.018	25.196	*p* < 0.001
PEU → PU	0.265	0.265	0.032	8.323	*p* < 0.001
PU → ATU	0.371	0.371	0.023	16.348	*p* < 0.001
PEU → ATU	0.211	0.212	0.025	8.300	*p* < 0.001
ECL → PU	−0.054	−0.054	0.009	5.865	*p* < 0.001
ICL → PU	−0.233	−0.233	0.023	10.292	*p* < 0.001
GCL → PU	0.451	0.451	0.026	17.628	*p* < 0.001
ECL → ATU	−0.181	−0.182	0.023	7.816	*p* < 0.001
ICL → ATU	−0.356	−0.356	0.026	13.698	*p* < 0.001
GCL → ATU	0.426	0.426	0.020	21.324	*p* < 0.001
ECL → PEU	−0.202	−0.202	0.025	8.190	*p* < 0.001
GCL → PEU	0.546	0.546	0.024	23.158	*p* < 0.001

Therefore, interventions aimed at increasing undergraduates’ behavioral intention to adopt SPES should prioritize the design of exercise experiences that actively foster schema construction, effective cognitive processing, and sustained engagement.

Although reducing unnecessary interface burden remains important, the findings suggest that interventions focusing exclusively on minimizing ICL and ECL may be insufficient unless they simultaneously enhance GCL (Table 12).

## 5 Discussion

This study integrates TAM and CLT to explain undergraduates’ BI to adopt SPES for EPA. The results show that BI is jointly shaped by technology-related evaluations and the allocation of limited cognitive resources during system use. Consistent with TAM, PEU, PU, and ATU functioned as central predictors of BI, whereas CLT revealed that GCL facilitated adoption and ICL and ECL suppressed intention. The integrated CL–TAM framework therefore provides a theoretically informed explanation for technology adoption in digitally mediated physical activity contexts.

Taken together, the findings indicate that undergraduates’ adoption of SPES for EPA is associated not only with the system’s functional characteristics, but also with how cognitive resources are allocated during system use. In particular, adoption is promoted when cognitive resources are directed toward meaningful processing and schema construction, but suppressed when they are consumed by task complexity or redundant information. Thus, the present study extends TAM by demonstrating that the effects of PEU, PU, and ATU are themselves contingent upon underlying cognitive load processes.

Regarding BI, the results further confirm that PEU remains the primary determinant (O’Dea, 2025). The magnitude of its path coefficient indicates that perceived ease of use is critical for sustaining engagement with SPES. This finding differs from much of the traditional TAM literature, in which PU is often regarded as the strongest determinant of BI (García et al., 2019). In the context of EPA, however, undergraduates may prioritize whether SPES can be used conveniently within fragmented and voluntary exercise situations. Therefore, the relative importance of PEU appears to increase when technology use is discretionary rather than mandatory (Brown et al., 2002).

In addition, both ATU and PU exert significant positive effects on BI, suggesting that evaluative judgments and perceived functional value among undergraduates also shape adoption decisions. These results are consistent with prior research across educational technology, digital health, and fitness applications, where higher PEU reliably predicts stronger BI (Cho et al., 2020; Vinnikova et al., 2020; Park et al., 2025). Moreover, PU is frequently identified as a key antecedent of BI, whereas ATU functions as an essential mediating mechanism linking cognitive evaluations to actual usage decisions (Alshammari and Babu, 2025). Evidence from Chinese university populations similarly shows that PU and ATU exert stable and substantial influences on BI, often explaining more variance than usability perceptions alone (Hai et al., 2022). However, prior evidence indicates that the effects of TAM core variables on BI are not fully consistent across contexts. For example, Songkram et al. (2023) reported a negative association between PU and BI, suggesting that task demands and usage pressure can undermine undergraduates’ subjective experiences. These results imply that the strength and direction of technology acceptance pathways are contingent on contextual and cognitive conditions.

Against this backdrop, the results highlight the distinctive role of GCL in shaping undergraduates’ BI to adopt SPES. When cognitive resources are invested in meaningful processing—such as understanding, integrating, and interpreting exercise-related information—BI increases. Prior research similarly shows that effort directed toward comprehension and schema construction promotes more favorable usage decisions and sustained engagement (Costley and Lange, 2017). Similarly, GCL strengthens BI primarily by enhancing PEU and other positive system evaluations, thereby facilitating adoption through indirect cognitive pathways (Gkintoni et al., 2025). More importantly, this finding suggests that cognitive load should not be viewed as uniformly detrimental in technology adoption research (Burnay et al., 2024). Whereas ICL and ECL consume limited cognitive resources, GCL facilitates adoption by directing those resources toward meaningful processing and schema construction. This interpretation aligns with Clarky and Kimmons (2023), who conceptualize germane load as productive cognitive effort rather than cognitive burden, supporting deeper processing and sustained participation. Moreover, recent evidence indicates that GCL serves as an important mediator between instructional design factors and BI, further underscoring its critical role in linking cognitive processes with technology adoption outcomes (Huang et al., 2025). In contrast, this study indicates that ICL exerts a significant inhibitory effect on university undergraduates’ BI to adopt SPES for EPA. When the inherent complexity of the exercise tasks consumes a substantial portion of undergraduates’ limited cognitive resources, their capacity for rational processing and value evaluation becomes constrained, thereby hindering the formation of positive usage decisions (Zucchelli et al., 2025). This result implies that optimizing interface design alone may be insufficient to improve undergraduates’ acceptance of SPES. Even a well-designed system may fail if the exercise tasks themselves remain cognitively overwhelming (Feltovich et al., 2004).

Further path analyses suggest that the influence of ICL on BI is not limited to a direct suppressive effect. Rather, ICL indirectly reduces BI by weakening undergraduates’ perceptions of the PU and their ATU. This study is consistent with the results reported by Huang et al. (2025), in the context of ChatGPT-based educational applications, observed that higher levels of intrinsic cognitive load significantly decreased undergraduates’ willingness to use the system, primarily through negative effects on attitudinal evaluations that subsequently influenced BI. This study also indicates that ECL exerts a significant negative influence on university undergraduates’ BI to adopt SPES for EPA. However, this result is not entirely consistent with prior research. For example, Huang et al. (2025), in the context of ChatGPT-based educational applications, reported that ECL did not have a significant effect on BI. This discrepancy may be attributed to the characteristics of EPA as a highly voluntary and context-dependent usage scenario, in which students typically complete exercise tasks under constraints of limited time and cognitive resources (Wintle, 2022; Antunes et al., 2024). As SPES become increasingly prevalent in higher education, students tend to develop baseline expectations regarding basic system functionality (Keane et al., 2023). Consequently, whether the system provides a clear and low-interference interaction experience may become a critical determinant of BI, because interface clarity supports perceived ease of use, whereas poor interface design can increase extraneous cognitive load and undermine usability perceptions (Faudzi et al., 2024).

When system interfaces are complex, information presentation is redundant, or operational procedures are unclear, undergraduates must allocate limited cognitive resources to processing information unrelated to their exercise goals (Sweller, 2024). This diversion of attention reduces their ability to fully recognize the system’s practical value in supporting extracurricular exercise (Yin and Sun, 2026). As cognitive evaluations deteriorate, undergraduates’ overall ATU becomes more negative, which ultimately suppresses their intention to continue using SPES for EPA (Schepers and Wetzels, 2007). Therefore, ECL may exert a broader influence on adoption than previously assumed because its effects extend beyond immediate usability perceptions and gradually undermine overall attitudes toward the system. Practically, the findings suggest that SPES should be designed to minimize unnecessary interface complexity while simultaneously reducing the intrinsic difficulty of exercise tasks. Exercise plans may need to be broken into smaller and progressively structured steps, particularly for inexperienced users (Gkintoni et al., 2025). In addition, personalized feedback, adaptive difficulty adjustment, and clear visual guidance may help direct cognitive resources toward meaningful processing, thereby increasing GCL and ultimately strengthening BI.

## 6 Implications

### 6.1 Theoretical implications

This study advances the theoretical understanding of undergraduates’ BI to adopt SPES by incorporating cognitive resource allocation into the analysis of technology acceptance. Although prior research has consistently demonstrated the robustness of TAM, the strength and direction of its core relationships often vary across contexts. This study demonstrates that such variability may stem less from limitations of TAM and more from differences in how cognitive resources are distributed and constrained during system use.

Integrating cognitive load constructs clarifies the conditions under which technology acceptance pathways operate effectively, particularly whether undergraduates possess sufficient cognitive capacity to form favorable perceptions of ease of use and usefulness. By differentiating among germane, intrinsic, and extraneous cognitive load, this study identifies distinct mechanisms through which each type influences adoption outcomes and extends the application of CLT to the domain of technology adoption research.

The results indicate that the formation of BI depends not only on the amount of cognitive effort invested but also on the direction of that effort. GCL enhances key cognitive evaluations by promoting meaningful processing, comprehension, and schema construction, whereas intrinsic and extraneous cognitive loads occupy or disrupt limited cognitive resources due to task complexity and system design burdens, thereby weakening the positive effects of technology acceptance pathways. This study provides a more nuanced theoretical framework for explaining the heterogeneous effects of different types of cognitive load on technology adoption. Moreover, the study enriches the applicability of both TAM and CLT from a contextualized perspective. Compared with mandatory or institutionalized usage settings, EPA is characterized by voluntary participation and immediate interaction, which makes the influence of cognitive load on acceptance pathways more salient. Collectively, these insights suggest that explanations of technology adoption behavior should integrate specific usage contexts with underlying cognitive mechanisms, thereby enabling the development of more context-sensitive theoretical models.

### 6.2 Practical implications

This study provides clear practical implications for designers of SPES, educational administrators, and university physical education practitioners. The results indicate that PEU remains the most critical determinant of BI.

This suggests that system promotion should not rely solely on the addition of new functions but should instead prioritize interface simplicity, clarity of operational procedures, and intuitive interaction design. By reducing learning costs and lowering barriers to use, institutions can effectively enhance undergraduates’ initial acceptance and sustained engagement with the system. From a cognitive load perspective, system design should focus particularly on minimizing ECL. Complex interface layouts, redundant information presentation, and cumbersome operational steps consume limited cognitive resources and weaken undergraduates’ overall evaluations of the system. Accordingly, SPES development should follow a “load-reduction design” principle, such as simplifying functional hierarchies, optimizing information visualization, and providing clear feedback and guidance.

These strategies can reduce unnecessary processing demands and improve undergraduates experience. At the same time, the results highlight the importance of fostering GCL to enhance undergraduates’ understanding of and sense of control over the system. Beyond merely simplifying design, systems should incorporate training guidance, data interpretation support, and personalized feedback to encourage effective cognitive processing. Such supportive design can help undergraduates integrate exercise-related information and develop stable training strategies, thereby strengthening perceived usefulness and positive attitudes toward use. Given that EPA represents a highly voluntary context, technology adoption depends more on undergraduates’ subjective experiences than on institutional mandates. Therefore, universities should complement system implementation with motivational strategies, contextualized guidance, and ongoing behavioral support rather than relying solely on administrative enforcement. By simultaneously optimizing both system design and the usage environment, institutions can facilitate a shift from superficial or procedural use to sustained and meaningful participation.

### 6.3 Limitations and future research

Several limitations should be noted. First, the study employed a cross-sectional design to examine the structural relationships among CL–TAM variables. Although this design captures associations at a single time point, longitudinal approaches would enable future research to assess dynamic changes in cognitive load and BI throughout sustained SPES use. The analysis focused on undergraduates, a population characterized by relatively high levels of digital technology use and participation in physical activity. Applying the proposed model to other populations or educational stages may reveal differences in cognitive load and technology acceptance mechanisms across undergraduate groups and further enhance the external validity and generalizability of the framework.

## Data Availability

The raw data supporting the conclusions of this article will be made available by the authors, without undue reservation.
