# Supplementary material for: An integrated cognitive load–technology acceptance model for explaining behavioral intention to adopt Smart Physical Education Systems for extracurricular physical activity
Source: Front Psychol. 2026 May 11;17:1811374. doi: 10.3389/fpsyg.2026.1811374 (PMC13199169; doi:10.3389/fpsyg.2026.1811374)
Supplement: Supplementary file 1 [file Supplementary_file_1.DOCX]

Measurement Items of the Study Constructs

| Constructs | Item Code | Measurement Item |
| --- | --- | --- |
| PU | PU1 | The Smart Physical Education System is useful for my extracurricular physical activity. |
|  | PU2 | The Smart Physical Education System helps me better meet the requirements of extracurricular physical activity. |
|  | PU3 | Using the Smart Physical Education System improves my overall efficiency in extracurricular physical activity. |
|  | PU4 | The Smart Physical Education System helps me carry out extracurricular physical activity more purposefully through data recording and feedback. |
|  | PU5 | The Smart Physical Education System helps me more comprehensively monitor, record, and analyze my extracurricular physical activity and health status. |
| PEU | PEU1 | The interface and operation procedures of the Smart Physical Education System are clear and easy to understand. |
|  | PEU2 | It is easy to learn how to use the Smart Physical Education System to fulfill extracurricular physical activity requirements. |
|  | PEU3 | It is easy to become skillful in using the Smart Physical Education System to record or manage extracurricular physical activity data. |
|  | PEU4 | The Smart Physical Education System enables me to get started quickly and effectively complete extracurricular physical activity tasks. |
|  | PEU5 | When I encounter operational problems while using the Smart Physical Education System, I can quickly find solutions or obtain technical support. |
| ATU | ATU1 | I hold a positive attitude toward using the Smart Physical Education System. |
|  | ATU2 | The Smart Physical Education System makes extracurricular physical activity more interesting. |
|  | ATU3 | I like the idea of using the Smart Physical Education System for extracurricular physical activity. |
|  | ATU4 | Using the Smart Physical Education System is a good way to support extracurricular physical activity. |
|  | ATU5 | I believe that using the Smart Physical Education System for extracurricular physical activity is meaningful. |
|  | ATU6 | I believe that using the Smart Physical Education System makes me more willing to participate in extracurricular physical activity. |
| BI | BI1 | I will actively and continuously use the Smart Physical Education System. |
|  | BI2 | I hope to continue using the Smart Physical Education System in the future. |
|  | BI3 | I will recommend the Smart Physical Education System to my classmates and friends. |
|  | BI4 | I intend to continue using the Smart Physical Education System in the coming period. |
|  | BI5 | In the future, I will give priority to using the Smart Physical Education System for extracurricular physical activity. |
|  | BI6 | I believe that I can maintain the habit of using the Smart Physical Education System for extracurricular physical activity. |
| ICL | ICL1 | I need to think repeatedly in order to understand the requirements of extracurricular physical activity. |
|  | ICL2 | Understanding the requirements of extracurricular physical activity requires me to integrate multiple pieces of knowledge. |
|  | ICL3 | It is not easy for me to understand and master the requirements of extracurricular physical activity. |
|  | ICL4 | I need to invest considerable effort to understand the requirements of extracurricular physical activity. |
|  | ICL5 | The process of combining the requirements of extracurricular physical activity with the use of the Smart Physical Education System requires mental effort. |
|  | ICL6 | Without clear external explanation or guidance, it is difficult for me to independently understand the full requirements of extracurricular physical activity. |
|  | ICL7 | The requirements of extracurricular physical activity involve too many consecutive steps, and I need to invest considerable effort to avoid mistakes or omissions. |
|  | ICL8 | I need to remember exercise-related information, such as time, location, and check-in rules, in order to fulfill the requirements of extracurricular physical activity. |
|  | ICL9 | Because I lack relevant experience, understanding the requirements of extracurricular physical activity is challenging for me. |
| ECL | ECL1 | I find it difficult to form a clear overall understanding of the Smart Physical Education System. |
|  | ECL2 | The interface and operation design of the Smart Physical Education System are inconvenient to use. |
|  | ECL3 | The interface and operational design of the Smart Physical Education System make it difficult for me to quickly locate important information. |
|  | ECL4 | The interface and operational design of the Smart Physical Education System make it difficult for me to understand the relationships among different steps. |
|  | ECL5 | The interface and operational design of the Smart Physical Education System make it difficult for me to fully focus on extracurricular physical activity. |
|  | ECL6 | Irrelevant content in the Smart Physical Education System, such as pop-up advertisements and redundant information notifications, disrupts the smoothness of my use experience. |
|  | ECL7 | The exercise behavior recognition functions of the Smart Physical Education System, such as facial recognition and location check-ins, increase my burden when using it. |
|  | ECL8 | The feedback services of the Smart Physical Education System, such as complaint handling, online customer service, and offline service platforms, still require improvement. |
| GCL | GCL1 | I actively use the Smart Physical Education System and make an effort to understand the information it provides. |
|  | GCL2 | After using the system for a period of time, I have formed a relatively complete understanding of its functions. |
|  | GCL3 | When using the Smart Physical Education System for exercise, I actively analyze my own exercise process. |
|  | GCL4 | Based on the requirements of extracurricular physical activity and the data provided by the system, I am able to develop and carry out an exercise plan that suits me. |
|  | GCL5 | I adjust my exercise arrangements dynamically according to the data provided by the Smart Physical Education System, such as frequency, duration, and intensity, in order to optimize the exercise effect. |
|  | GCL6 | I am able to analyze the data from the Smart Physical Education System together with my physical condition indicators, such as body fat percentage, BMI, and blood pressure, in order to gain a more comprehensive understanding of my health status. |
